# Exceptional Thermoelectric Properties of Bilayer GeSe: First Principles Calculation

**DOI:** 10.3390/ma15030971

**Published:** 2022-01-27

**Authors:** Qiang Fan, Weibin Zhang, Haiyin Qing, Jianhui Yang

**Affiliations:** 1 School of New Energy Materials and Chemistry, Leshan Normal University, Leshan 614004, China; fq1893@foxmail.com; 2 Institute of Physics and Electronic Information, Yunnan Normal University, Kunming 650500, China; zmrright@163.com; 3 School of Electronic Information and Artificial Intelligence, Leshan Normal University, Leshan 614004, China; qinghaiyin123@163.com; 4 School of Mathematics and Physics, Leshan Normal University, Leshan 614004, China

**Keywords:** heterostructure, electronic structure, thermoelectric properties, GeSe, SnSe

## Abstract

The geometry structures, vibrational, electronic, and thermoelectric properties of bilayer GeSe, bilayer SnSe, and van der Waals (vdW) heterostructure GeSe/SnSe are investigated by combining the first-principles calculations and semiclassical Boltzmann transport theory. The dynamical stability of the considered structures are discussed with phonon dispersion. The phonon spectra indicate that the bilayer SnSe is a dynamically unstable structure, while the bilayer GeSe and vdW heterostructure GeSe/SnSe are stable. Then, the electronic structures for the bilayer GeSe and vdW heterostructure GeSe/SnSe are calculated with HSE06 functional. The results of electronic structures show that the bilayer GeSe and vdW heterostructure GeSe/SnSe are indirect band gap semiconductors with band gaps of 1.23 eV and 1.07 eV, respectively. The thermoelectric properties, including electrical conductivity, thermal conductivity, Seebeck coefficient, power factor, and figure of merit (*ZT*) are calculated with semiclassical Boltzmann transport equations (BTE). The results show that the *n*-type bilayer GeSe is a promising thermoelectric material.

## 1. Introduction

With the decline of fossil fuel reserves and the increase in environmental pollution caused by energy consumption, the research activities related to the development of alternative technologies that can use renewable energy have increased significantly. In addition, a lot of energy is wasted in the form of heat during the use of energy. Therefore, due to the Seebeck effect, thermoelectric materials that can convert waste heat directly into useful electrical energy have attracted great attention. The efficiency of thermoelectric conversion of thermoelectric materials is usually determined by the dimensionless figure of merit *ZT*, $ZT=\frac{S^{2}\sigma T}{k}$, where *S* is the Seebeck coefficient, *σ* represents electrical conductivity, and *k* is thermal conductivity including electron (*k_e_*) and lattice (*k_l_*) contribution. $S^{2}\sigma$ is commonly referred to the power factor (*PF*) of thermoelectric materials. Improving *ZT* value is the ultimate goal of researchers in the thermoelectric community. However, this goal is very difficult to achieve due to the strong coupling between physical parameters. For example, the carrier concentration has the opposite effect on Seebeck coefficient and electrical conductivity: the decrease in carrier concentration increases the Seebeck coefficient, but meanwhile, it leads to a decrease in electrical conductivity, and vice versa. On the other hand, toxic, high price, and low earth content are other factors restricting the development of thermoelectric materials [1,2].

Layered material, such as thallium oxygen, bismuth oxygen selenide, and tin chalcogenide, show great promise in thermoelectric applications due to the intrinsic low lattice thermal conductivity with phonon anharmonicity generated by the interaction between adjacent layers [3,4,5,6]. Since the discovery of excellent thermoelectric performance of single-crystal SnSe [7], the thermoelectric properties of nontoxic, earth-abundant group IV-VI have attracted special attention [8,9,10,11,12]. While the extraordinarily thermoelectric performance of SnSe benefits from its single-crystal crystallization, the thermoelectric performance for the polycrystalline sample is still far from being satisfactory [13,14]. Several approaches have been proposed to enhance the thermoelectric performance of bulk group IV–VI compounds, such as intrinsic vacancy [15], defect dopants [16], substitutional doping [17,18], alloying [19], nanostructuring [20], strain lattice [21], and textural microstructure [22] to modify electronic structure, enhance the carrier concentration, or reduce the thermal conductivity. The group IV–VI compounds possess a puckered two-layered structure, and the two layers are interconnected with weak van der Waals forces, while the atoms in the layer form strong covalent interaction. The adjacent layer structure makes it possible for the synthesis of a two-dimensional (2D) structure. In fact, the 2D nanosheets of SnSe and GeSe have been successfully synthesized [23,24]. In addition, theoretical study has found that the 2D structure of group IV–VI compounds are thermodynamically stable with effectively low lattice thermal conductivity [25]. Numerous theoretical studies have been revealed the thermoelectric performance of 2D group IV–VI compounds [26,27,28,29]. The results showed that 2D group IV–VI compounds show interesting thermoelectric performance and are suitable for renewable thermoelectric applications.

Stacking two 2D materials to construct a bilayer or van der Waals (vdW) heterostructure is an effective way to regulate the electronic structure and improve the thermoelectric properties of materials [30,31,32,33,34,35,36]. The SnSe/GeSe nanosheet heterojunction has been successfully prepared in the experiment by Sun et al. in 2017 [37]. It has been proved that two kinds of crystallographically aligned SnSe/GeSe nanosheet heterostructures can be produced. The upper and lower atoms form different relative positions, resulting in a variety of heterostructures. As reported by Mao et al. [35], the AA tacking—that is, when the top layer is directly stacked on the bottom layer without any relative rotation—of bilayer GeSe is the most stable. Theoretical studies by Ni et al. [38] indicated that the AA stacking of the SnSe/GeSe heterojunction has good stability. In this paper, we investigate the thermoelectric properties of homogeneous bilayer GeSe, SnSe, and vdW heterostructure GeSe/SnSe in an AA stacking model.

## 2. Computational Details

The first principle calculations are carried out with the help of the Vienna ab initio simulation package (VASP, version 5.4.4) using the projected augmented wave (PAW) method [39]. The exchange correlation functional used in this work is Perdew–Burke–Ernzerhof (PBE) and takes into account Hartree Fock exchange Hybrid Functional HSE06 [40]. The DFT-D2 method of Grimme is adopted to describe the van der Waals interaction [41]. In all calculations, a well-converged kinetic energy cutoff for the plane wave is set to 600 eV. The first Brillouin zone is sampled with a 12 × 12 × 1 k-point grid using the Monkhorst–Pack method. All structures are fully relaxed until the total energy and force are less than 10^−6^ eV/atom and 0.001 eV/Å, respectively. To prevent the fictitious interactions of the periodic boundary conditions, a large vacuum thickness over 20 Å is considered between adjacent layers in the perpendicular direction.

After obtaining the optimized structures, the vibrational properties are investigated to verify the dynamic stability of the structures. The phonon dispersion is calculated from harmonic interatomic force constants employed in PHONOPY code [42]. A 5 × 5 × 1 supercell with 3 × 3 × 1 k-point mesh is constructed to ensure the convergence. The lattice thermal conductivity is calculated by using the Slack model [43]. According to the Slack equation, the lattice thermal conductivity $k_{l}$ can be derived from

(1) $$k_{l}=A\frac{\bar{M}\theta_{D}^{3}\delta }{\gamma^{2}n^{2/3}T}$$

in which *A* is a constant equal to 3.04 × 10^−6^. The $\bar{M}$, *δ*, *n*, and *T* are the average atomic mass, cubic root of volume per atom, number of atoms in the primitive cell, and temperature, respectively. γ denotes the Grüneisen parameter, reflecting the anharmonicity of lattice oscillations and nonlinearity of interatomic forces, which can be calculated by longitudinal ($v_{l}$) and transverse ($v_{t}$) sound velocities [44,45]:

(2) $$\gamma =\frac{9\left(\nu_{l}^{2}-4\nu_{t}^{2}/3\right)}{2\left(\nu_{l}^{2}+2\nu_{t}^{2}\right)}$$

The Debye temperature ($\theta_{D}$) is associated with many physical properties of materials, which can be given as the following equation:

(3) $$\theta_{D}=\frac{h}{k_{B}}{\left(\frac{3n}{4\pi \Omega }\right)}^{1/3}v_{m}$$

where the *h*, *k_B_*, *n*, *ν_m_*, and Ω are the Planck constant, Boltzmann constant, number of atoms in the unit cell, average sound wave velocity, and cell volume, respectively.

Based on electronic structure, the transport properties of dynamic stable structures are investigated by solving a semiclassical Boltzmann transport equation (BTE) with the help of the BoltzTraP program [46]. When solving BTE, two approximations are used, that is, the approximations of constant relaxation time (CRT) and rigid band (RB). In the CRT approximation, the relaxation time (*τ*) of all electronic states has the same value, while in the RB approximation, doping will only lead to the shift of the chemical potential of the system, but it does not alter the energy band dispersion. Due to the complex scattering mechanism in materials, it is difficult to obtain the relaxation time accurately. In fact, for semiconductors, the *τ* is typically of the order of femtosecond (fs). In this work, the *τ* is adopted as 10 fs for all materials, which is practicable [33,47]. In addition, a denser k-point mesh helps to obtain more reliable thermoelectric transport properties. For this purpose, in this process, a much denser k-point mesh of 25 × 25 × 1 is used in the self-consistent calculation of electronic structure.

## 3. Results and Discussion

### 3.1. Geometry Optimization and Electronic Structure

**Figure 1:**
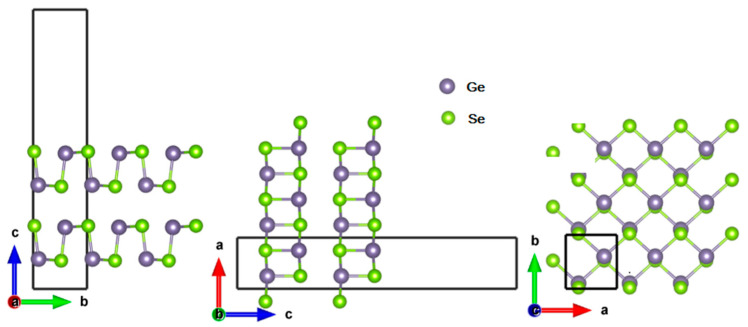
The side views of the structure of bilayer GeSe.

In the bulk case, both GeSe and SnSe are double-layer orthorhombic structures with a Pnma (No.62) space group. However, the monolayer GeSe and SnSe stripped from the bulk structures belong to the Pmn2_1_ (No. 31) space group. The monolayer structures of GeSe and SnSe are fully optimized. The optimized lattice parameters of monolayer GeSe and SnSe are 3.98 (4.29) Å and 4.30 (4.40) Å along zigzag (armchair) directions, which are in good agreement with the previous reported values 3.96 (4.22) Å [35] and 3.94 (4.30) Å [48] for monolayer GeSe and 4.30 (4.34) Å for monolayer SnSe [29]. Based on the optimized 2D monolayer GeSe and SnSe, the homogeneous bilayer GeSe, bilayer SnSe, and vdW heterostructure GeSe/SnSe are constructed. The side views of the structure of bilayer GeSe are shown in Figure 1. When constructing heterostructure GeSe/SnSe, the lattice mismatches for monolayer GeSe and monolayer SnSe are 1.03% and 0.95%, respectively, which can be well matched in the establishment of a heterojunction.

The optimized lattice constants along the zigzag (armchair) direction are 3.93 (4.14) Å, 4.29 (4.39) Å, and 4.06 (4.51) Å for the bilayer GeSe, bilayer SnSe, and heterostructure GeSe/SnSe, which are in good agreement with other existing first-principles calculations [35,36]. The interlayer distance is defined as the minimum vertical distance between the upper and lower layers. The optimized interlayer distances of the bilayer GeSe, bilayer SnSe, and heterostructure GeSe/SnSe are 3.15 Å, 3.50 Å, and 2.99 Å, respectively. The interlayer distances are compared with the sum of the van der Waals radius of the atoms, which indicates no bonding between the two monolayers, and van der Waals interactions hinge them together.

### 3.2. Stability, Lattice Thermal Conductivity

**Figure 2:**
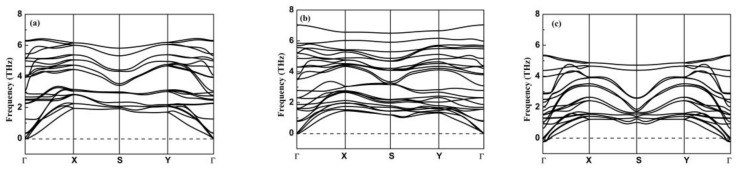
Phonon dispersion curve of bilayer GeSe (**a**), heterostructure GeSe/SnSe (**b**), and bilayer SnSe (**c**).

To check the dynamical stability of the optimized structures, the phonon dispersion is calculated and analyzed. Figure 2 shows the calculated phonon dispersion curves of bilayer GeSe, bilayer SnSe, and heterostructure GeSe/SnSe along the high-symmetry path of k-points.

We note that there are 24 phonon modes, including three acoustic branches and 21 optical branches. In the first Brillouin zone, there is no imaginary frequency in the phonon dispersion of bilayer GeSe and heterostructure GeSe/SnSe. However, an obvious imaginary frequency of the acoustic branches appears near the Γ point in the bilayer SnSe phonon dispersion. These results suggest that the structures of bilayer GeSe and heterostructure GeSe/SnSe are dynamically stable, while bilayer SnSe is dynamically unstable. The previous first-principles calculation indicates that AA stacking of bilayer SnSe is not the most stable, either [49]. Therefore, in this work, we do not further study the electronic and thermoelectric properties of bilayer SnSe. With the phonon dispersion, the phonon group velocity v, Debye temperature $\theta_{D}$, and Grüneisen parameters γ for bilayer GeSe and heterostructure GeSe along the zigzag and armchair direction are calculated, and the results are tabulated in Table 1.

**Figure 3:**
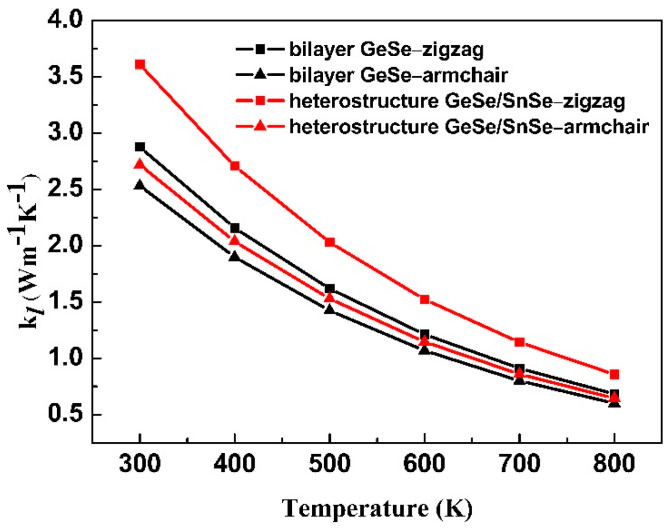
The calculated lattice thermal conductivity as a function of temperature.

The Grüneisen parameters along the zigzag direction are larger than those along the armchair direction for both bilayer GeSe and heterostructure GeSe/SnSe. We further calculate the lattice thermal conductivity of bilayer GeSe and heterostructure GeSe/SnSe along the zigzag and armchair directions at different temperatures estimated with Equation (1). The calculated lattice thermal conductivity as a function of temperature is exhibited in Figure 3.

The lattice thermal conductivity along the zigzag and armchair directions are anisotropic with the value along the armchair direction being smaller than that along the zigzag direction. With the increase in temperature, the thermal conductivity decreases. At 300 K, the calculated lattice thermal conductivity along the zigzag (armchair) direction of the bilayer GeSe and heterostructure GeSe/SnSe are 2.88 (2.53) Wm^−1^K^−1^ and 3.61 (2.72) Wm^−1^K^−1^, respectively. The lattice thermal conductivity of the bilayer GeSe and heterostructure GeSe/SnSe along different directions has the same trend as those of the monolayer GeSe and SnSe [50]. The lattice thermal conductivity of the bilayer GeSe is lower than the corresponding values of monolayer GeSe (6.7 Wm^−1^K^−1^ and 5.2 Wm^−1^K^−1^ along the zigzag and armchair direction, respectively). In contrast, the lattice thermal conductivity of the heterostructure GeSe/SnSe is close to that of monolayer SnSe (2.6 Wm^−1^K^−1^ and 2.4 Wm^−1^K^−1^ along the zigzag and armchair direction, respectively) [26]. 

**Figure 4:**
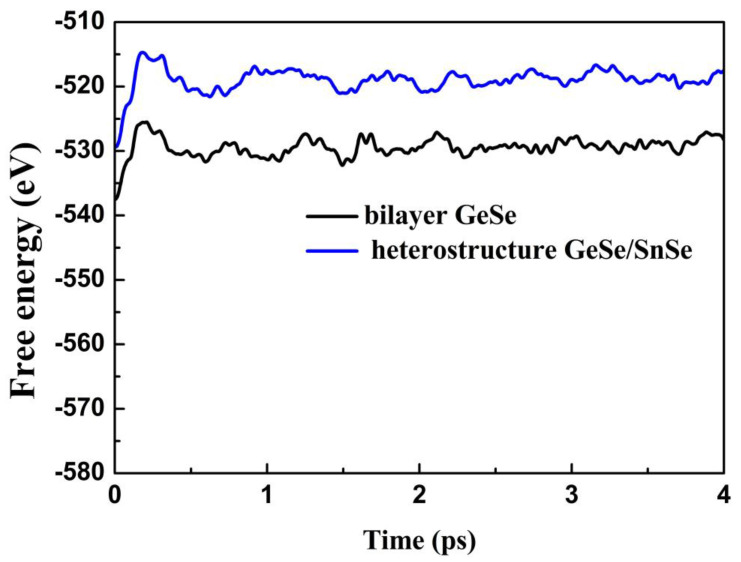
Free energy fluctuations with respect to time in AIMD simulations at 700 K for the bilayer GeSe and heterostructure GeSe/SnSe.

To study the thermal stability of the bilayer GeSe and heterostructure GeSe/SnSe at high temperature, we perform ab initio molecular dynamics (AIMD) simulations at 700 K. A 4 × 4 × 1 supercell is constructed to minimize the constraint induced by periodicity. The simulated results at 700 K are given in Figure 4. We find that the average values of the total energy remain almost invariant after 0.5 ps both for the bilayer GeSe and heterostructure GeSe/SnSe. Therefore, the structures are thermally stable at 700 K. Consequently, we consider up to 700 K as the typical temperature to study the thermoelectric properties of the bilayer GeSe and heterostructure GeSe/SnSe.

### 3.3. Electronic Structure

**Figure 5:**
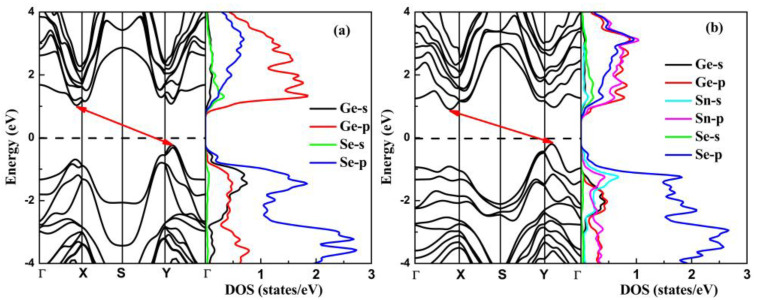
The band structures and PDOS for the bilayer GeSe (**a**) and heterostructure GeSe/SnSe (**b**). The Fermi level is set at 0 eV. The arrow highlights the CBM and VBM positions.

The electronic properties of the stable bilayer GeSe and heterostructure GeSe/SnSe are analyzed. The electronic band structures along the high-symmetry path of k-points for the bilayer GeSe and heterostructure GeSe/SnSe calculated with the HSE06 function are shown in Figure 5. To further clarify the electronic structures, the corresponding partial density of states (PDOS) is also presented together in Figure 5.

As shown in Figure 5, the conduction band appears at two similar local extrema respectively at (0, 0.42, 0) along the Γ-Y direction and (0.42, 0, 0) along the Γ-X direction in the electronic band structures for the bilayer GeSe and heterostructure GeSe/SnSe. The calculated results show that the energy of the (0, 0.42, 0) point is only 0.05 and 0.07 eV larger than that of the (0.42, 0, 0) point, respectively, for the bilayer GeSe and heterostructure GeSe/SnSe. Thus, both the bilayer GeSe and heterostructure GeSe/SnSe have indirect band gaps, and the band gap values are 1.23 eV and 1.07 eV, respectively. The calculated band gap of the bilayer GeSe is consistent with other HSE06 calculations [35]. Meanwhile, from the PBE function, the energy band gap of the heterostructure GeSe/SnSe was predicted as only 0.29 eV [38]. The valence band maximum (VBM) and conduction band minimum (CBM) for the GeSe and SnSe layers are also located along the Y-Γ and Γ-X paths [51]. In addition, because the difference between the two local extrema of the conduction band is very small and the CBM is easy to switch, the layer GeSe are usually regarded as nearly direct band gap semiconductors [52]. In order to further understand the electronic properties of the above structures, in Figure 5, we also present the PDOS to provide more insights into the contribution of atomic orbitals. We can clearly see that the p orbital of the Se atoms show mainly the contribution of the total DOS in the upper valence band region for the bilayer GeSe and heterostructure GeSe/SnSe. Above the Fermi level, in the bottom valence band region, the p orbital of the Ge atom mainly contributes to the total DOS of the bilayer GeSe, while the major component of total DOS for the heterostructure GeSe/SnSe are the p orbitals of the Ge, Sn, and Se atoms.

### 3.4. Electronic Transport and Thermoelectric Properties

**Figure 6:**
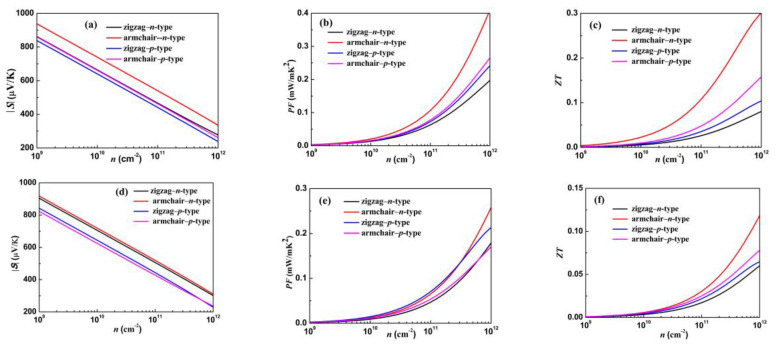
The transport coefficients of the bilayer GeSe (Seebeck coefficient (**a**), power factor (**b**), and *ZT* (**c**)) and heterostructure GeSe/SnSe (Seebeck coefficient (**d**), power factor (**e**), and *ZT* (**f**)) along the zigzag and armchair directions as a function of carrier concentration (*n*) at 300 K.

Based on the calculated electronic structures, the electronic transport properties are investigated with Boltzmann transport theory. The electronic transport properties including the Seebeck coefficient (*S*), power factor (*PF*), and *ZT* value as a function of the carrier concentration (*n*) for the bilayer GeSe and heterostructure GeSe/SnSe along the zigzag and armchair directions at 300 K are illustrated in Figure 6. The Seebeck coefficient has a negative value for the *n*-type compound, and for the *p*-type compound, the Seebeck coefficient value is positive; we show the absolute value of *S* (|S|) in Figure 6.

For promising application in thermoelectric devices, the |S| is usually required to be larger than 200 μV/K. A remarkable Seebeck coefficient can be obtained with smaller *n*-type or *p*-type doping both for the bilayer GeSe and heterostructure GeSe/SnSe. The Seebeck coefficients along the zigzag and armchair directions both for bilayer GeSe and heterostructure GeSe/SnSe display anisotropy due to the weak symmetry band structure. We note that *n*-type doping has a larger Seebeck coefficient than *p*-type one for the heterostructure GeSe/SnSe, which can be explained by the high contrast of DOS, especially near the edge of the band gap (see Figure 5b). It is worth noting that the *n*-type bilayer GeSe along the armchair direction shows a significantly large Seebeck coefficient, which can be attributed to the conduction band convergence. A local extrema of the conduction band is located along the armchair direction with only 0.05 eV smaller than CBM, which is beneficial to improve the band degeneracy along the armchair direction. The BoltzTrap code calculates the electrical conductivity and electronic thermal conductivity within constant *τ* approximation, i.e., *σ*/*τ* and *κ*_e_/*τ*. A typical *τ* = 10 fs is used to obtain electrical conductivity and electronic thermal conductivity. Since *σ* is larger at higher *n*, while *S* has a larger value at lower *n*, therefore, it is necessary to balance *σ* and *S* to have a remarkable *PF*. As can be clearly seen, the *PF* shows obvious anisotropy when the carrier concentration is higher than 1 × 10^10^ cm^−2^. In the considered range, the *PF* increases rapidly with the increase for both electrons and holes, which is attributed to the rapid increase in electrical conductivity with carrier concentration. In both *n*-type and *p*-type bilayer GeSe, the *PF* along the armchair direction is greater than that along the zigzag direction. In *n*-type heterostructure GeSe/SnSe, the *PF* along the armchair direction is greater than that along the zigzag direction; however, it is the opposite trend in *p*-type heterostructure GeSe/SnSe. Even more remarkably concerning, the *PF* along the armchair direction of *n*-type bilayer GeSe is significantly larger than that along the zigzag direction for the significantly large Seebeck coefficient. The electronic thermal conductivity is related to electrical conductivity with the Wiedemann–Franz law: $k_{e}=L\sigma T$. So, the electronic thermal conductivity has a similar trend to electrical conductivity. Finally, the figure of merit *ZT* is obtained with the calculated transport coefficients and lattice thermal conductivity. At room temperature, the figure of merit *ZT* shows anisotropic behavior and increases rapidly with carrier concentration in the considered doping level, which keeps pace with *PF*. The *ZT* value along the armchair direction is much larger than that along the zigzag direction for the bilayer GeSe and heterostructure GeSe/SnSe, which can be attributed to the lower lattice thermal conductivity along the armchair direction. We find that the *n*-type bilayer GeSe along the armchair direction shows excellent thermoelectric performance.

**Figure 7:**
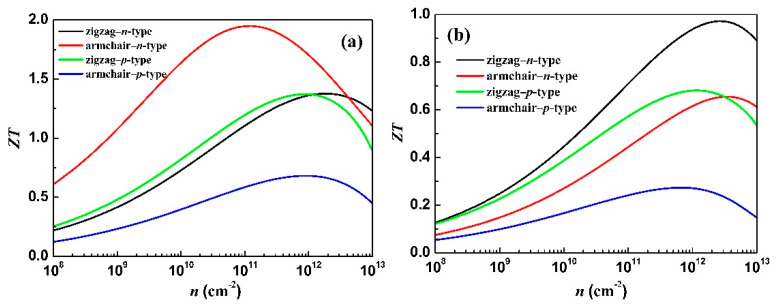
Calculated figures of merit (*ZT*) of the bilayer GeSe (**a**) and heterostructure GeSe/SnSe (**b**) along the zigzag and armchair directions as a function of the carrier concentration (*n*) at 700 K.

Generally, doping is beneficial to *σ*, while it deteriorates *S*. Thus, the *ZT* first increases and then decreases with the increase in carrier concentration, resulting in the optimal *ZT*. In addition, the *ZT* value invariably increases with the increase in temperature. The calculated figures of merit *ZT* of bilayer GeSe and heterostructure GeSe/SnSe along the zigzag and armchair directions as a function of the carrier concentration at 700 K are shown in Figure 7.

At 700 K, the *ZT* maxima of bilayer GeSe are 1.37 (1.37) along the zigzag and 1.95 (0.68) along the armchair directions in the optimal electron (hole) doping. The peak values of ZT are 0.97 (0.68) along the zigzag and 0.66 (0.27) along the armchair directions, respectively, in the optimal electron (hole) doping for heterostructure GeSe/SnSe. We find the peak *ZT* value for *n*-type bilayer GeSe along the armchair direction occurs at the electron concentration of 1.29 × 10^11^ cm^−^^2^. Such a low doping concentration is easy to obtain. The high *ZT* value along the armchair direction for *n*-type bilayer GeSe suggests that bilayer GeSe can be a promising candidate for applications in thermoelectric generators based on a proper electron doping level.

## 4. Conclusions

The electronic structure and thermoelectric properties of AA-stacked bilayer GeSe and heterostructure GeSe/SnSe are investigated by using the first-principle calculation combined with Boltzmann transport theory. The bilayer GeSe and heterostructure GeSe/SnSe are indirect band gap semiconductors with band gaps of 1.23 and 1.07 eV, respectively. The bilayer GeSe and heterostructure GeSe/SnSe have lower lattice thermal conductivity in comparison with layer GeSe and SnSe. The thermoelectric properties for the bilayer GeSe and heterostructure GeSe/SnSe display anisotropy. The *n*-type bilayer GeSe along the armchair direction shows a significantly large Seebeck coefficient. The peak values of *ZT* along the zigzag and armchair directions are 1.37 (1.37) and 1.95 (0.68) in the *n* (*p*) -type bilayer GeSe at 700 K. Meanwhile, the *ZT* maxima of the heterostructure GeSe/SnSe in the optimal electron (hole) doping along the zigzag and armchair directions are 0.97 (0.68) and 0.66 (0.27), respectively. Therefore, the *n*-type bilayer GeSe is a promising thermoelectric material. We expect that the nanostructured bilayer GeSe can be applied in a nanoscale thermoelectric device, and it can obviously promote a nanoscale thermoelectric device for practical application.

## Figures and Tables

**Table 1:** The calculated phonon group velocity *ν* (m/s), Debye temperature $\theta_{D}$ (K), and Grüneisen parameters γ.

Material	Direction	*ν* (m/s)			*γ*	$\theta_{D}$ (K)
		ZA	TA	LA		
GeSe	zigzag	592	778	1425	1.698	183
	armchair	249	452	855	1.810	183
GeSe/SnSe	zigzag	626	870	1591	1.694	188
	armchair	246	583	1149	1.952	188

## Data Availability

Data available on request. The data presented in this study are available on request from the corresponding author.
